# Mushroom against Cancer: Aqueous Extract of *Fomitopsis betulina* in Fight against Tumors

**DOI:** 10.3390/nu16193316

**Published:** 2024-09-30

**Authors:** Paulina Nowotarska, Maciej Janeczek, Benita Wiatrak

**Affiliations:** 1 Department of Biostructure and Animal Physiology, Wroclaw University of Environmental and Life Sciences, Norwida 25/27, 50-375 Wroclaw, Poland; maciej.janeczek@upwr.edu.pl; 2 Department of Pharmacology, Wroclaw Medical University, Mikulicza-Radeckiego 2, 50-345 Wroclaw, Poland

**Keywords:** *Fomitopsis betulina*, in vitro, anticancer activity, plant model organisms, cytotoxicity

## Abstract

**Background/Objectives:** This study investigated the anticancer potential of an aqueous extract of the fungus *Fomitopsis betulina*. **Methods:** The study assessed the effect of the extract on nine cancer cell lines, including melanoma (LM-MEL-75), lung cancer (A549), and colorectal cancer (HT29, LoVo), and four normal cell lines. The cytotoxicity of the extract was evaluated using MTT, sulforhodamine-B (SRB), and clonogenic viability assays. Additionally, the study examined the effect of the extract on plant model organisms, garden cress (*Lepidium sativum*) and common onion (Allium cepa), to further investigate its biological activity. **Results:** The assays demonstrated selective cytotoxicity of the extract toward cancer cells, while sparing normal cells. The extract induced significant cytotoxic effects at lower concentrations in lung cancer, melanoma, and colon cancer cells, showing promise as a potential anticancer agent. The results also revealed that the extract inhibited seed germination and root growth, suggesting its potential to disrupt cell cycles and induce apoptosis. **Conclusions:** This study highlights the therapeutic potential of *F. betulina* and highlights the need for further research to identify the active ingredients and mechanisms underlying its anticancer effects.

## 1. Introduction

Cancer poses a significant challenge for modern medicine, necessitating continuous research to discover new and effective therapies. In vitro studies on compounds of both plant and fungal origin play a crucial role in identifying potential anticancer drugs. These studies allow for precise assessments of how these compounds impact the growth, proliferation, and survival of cancer cells.

Plant compounds, abundant in nature, have long intrigued researchers due to their potential anticancer properties. Flavonoids, alkaloids, polyphenols, and other phytochemicals found in many plants exhibit anticancer activity [1]. In vitro investigations enable us to understand the cellular mechanisms of action of these compounds, aiding in the selection of promising candidates for further clinical research [2]. Fungi, with their genetic and metabolic diversity, represent another promising source of potential anticancer compounds. In recent years, scientists have focused on identifying biologically active fungal compounds for cancer therapy [3]. *Fomitopsis betulina* (formerly known as *Piptoporus betulinus*) has garnered scientific interest due to its potential health benefits. However, clinical research on this mushroom remains limited, and preclinical studies have shed light on its promising properties. *Fomitopsis betulina* is commonly found on birch trees. An intriguing historical connection lies in the mummified remains of Ötzi (the Iceman), who lived approximately 5300 years ago. Ötzi carried fragments of *Fomitopsis betulina*, which is commonly found on birch trees, suggesting its use for medicinal purposes even in ancient times [4].

It occurs in the northern hemisphere [5] and is considered a common species in Poland. It grows on both living tree trunks and dead wood fragments as well as on branches. The birch polypore exclusively grows on trees from the birch family, such as silver birch, downy birch, dark birch, and paper birch [4,6]. Its presence spans from August to October, and the dead fruiting bodies remain on the tree throughout the year [7]. However, attention must be paid to the location of the trees from which the fungus is collected as environmental pollutants in industrial zones may lead to contamination with heavy metals and toxins. Such factors have been known to impact the chemical composition and medicinal properties of fungi.

This fungus is an annual organism. After penetrating a wound site in healthy birch bark, it can remain in a state of hibernation for many years. Interestingly, studies on other medicinal fungi, such as *Ganoderma lucidum* and *Trametes versicolor*, have noted similar resilience and adaptive strategies in nature, which may contribute to their therapeutic effects [8,9]. Only when the tree weakens (due to excessive shade, drought, or fire) does the birch polypore activate, breaking down wood and causing white rot—a feeding strategy that classifies it as a parasite [10]. The cap of the birch polypore is semicircular and gray-brown, droops 3–15 cm, has a width of 4.5–12.5 cm, and has a thickness of 1.7–3.5 cm at the base. The mushroom tubes reach a length of up to 8 mm, and in mature specimens, they easily detach from the cap flesh [5]. In mature specimens, the hymenium appears white to ocher, and the pores are round or angular and occur at a rate of 3–5 per millimeter. Initially, they are thick and filled with secretions, but over time, they rupture, tear, and eventually develop a hydnoid structure. The tube layer can be easily separated from the surrounding context and may reach a thickness of up to 10 mm [5]. The flesh is white, bitter, and astringent in taste. Some sources suggest that young specimens are edible [5,11].

The infusion of *F. betulina* basidiom gained popularity, particularly in Russia, the Baltic countries, Hungary, and Romania, for its calming effects, nutritional benefits, and anti-fatigue properties [12,13,14]. This is consistent with the use of other medicinal mushrooms such as Chaga in Siberian and Baltic traditional medicine, which also offers antioxidant and immune-modulating properties [15]. Additionally, mushroom tea served as an antiparasitic remedy and was used to address gastrointestinal issues [12,13,14]. In the Czech Republic, *Fomitopsis betulina* was utilized for treating rectal cancer and stomach ailments [13]. Interestingly, the polypore fungus has been recognized for its antiseptic and analgesic properties in both Europe and the USA [13,16]. Traditionally, *F. betulina* has been employed as an antiparasitic and antimicrobial agent to address wounds and control bleeding [17]. Fresh fruiting bodies of *F. betulina* were used to create antiseptic and anti-bleeding dressings, while the powder derived from dried material served as a painkiller [12,18,19]. Additionally, it found use as tinder and an anesthetic [20]. *Fomitopsis betulina* has exhibited antibacterial activity, particularly against Gram-positive bacteria such as *Streptococcus* and *Staphylococcus*. It is also used in folk medicine, especially in Baltic countries, due to its antifungal and antiparasitic properties [11,20]. The specific active compounds responsible for this effect are still under investigation. Additionally, preparations derived from *Fomitopsis betulina* have shown immunomodulatory effects and potential neuroprotective properties.

In vitro studies have revealed that extracts from *Fomitopsis betulina* contain triterpenes and polysaccharides, which exhibit anticancer activity by inducing apoptosis, inhibiting angiogenesis, and blocking cell signaling pathways. These discoveries suggest that *F. betulina* could be a valuable component or source of anticancer drugs. In particular, the presence of triterpenes, similar to those found in *Ganoderma* and Chaga mushrooms, may explain the apoptotic and cytostatic effects observed in cancer cell lines [15,21]. While clinical trials involving this fungus are scarce, there is ongoing research that aims to explore its potential applications in health and biotechnology.

Previous studies have investigated the anticancer properties of ethanolic extracts of *F. betulina*, mainly focusing on ethanolic extracts [8,9]. This study aims to address this gap by investigating the effect of an aqueous extract from *F. betulina* on a panel of cancer cell lines.

In this context, the current study seeks to evaluate the selective cytotoxicity of aqueous extracts from *Fomitopsis betulina* on several cancer cell lines while also assessing its effect on normal cell lines. Additionally, we explore the biological activity of the extract using plant model organisms, such as *Lepidium sativum* (garden cress) and *Allium cepa* (onion), to assess its broader biological impact. This research contributes to the growing body of evidence on natural extracts in cancer therapy, offering insights into the therapeutic potential of aqueous extracts that may be safer than alcohol-based alternatives.

## 2. Results

### 2.1. Viability Assays

#### 2.1.1. MTT Assay

**Figure 1:**
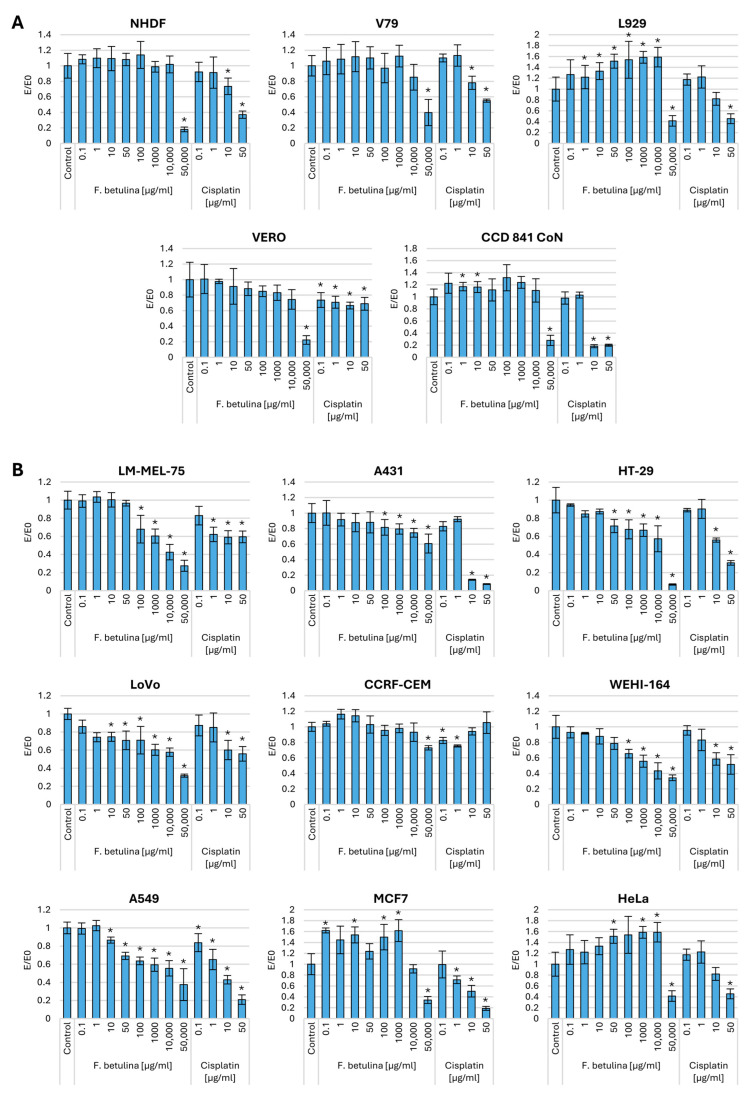
Mitochondrial activity after incubation with aqueous extract of *F. betulina*: (**A**) Normal cell lines; (**B**) Cancer cell lines. Data are presented as E/E_0_ (where E—the average number of colonies in the tested extract concentration and E_0_—the average number in the control culture and standard deviation (SD); * statistically significant differences in mitochondrial activity (*p* < 0.05) when comparing treated versus control groups, with error bars representing standard deviation.

To investigate the impact of the aqueous extract of *F. betulina* on the cytotoxic potential of cancer cells, cell viability studies were conducted by incubating the extract for 24 h using the MTT assay on nine cancer cell lines. The MTT test allows the assessment of mitochondrial activity in cells. Figure 1 illustrates the effect of the extract on cells pre-incubated with either the tested extract or cisplatin (a chemotherapeutic agent commonly used in cancer treatment). Traditional cytostatics are highly toxic not only to cancer cells but also to normal cells. Therefore, the influence of the tested extract was also evaluated on four normal cell lines. The study revealed that the extract, up to a concentration of 10,000 µg/mL, did not exhibit cytotoxic potential in normal mice (L929), hamsters (V79), and human (NHDF) fibroblasts. Moreover, in the case of the mouse fibroblast line, in the concentration range of 1–10,000 ug/mL, a significant increase in mitochondrial activity was observed compared to the control. In contrast, cisplatin demonstrated cytotoxicity in human and mouse fibroblast lines at a concentration as low as 10 µg/mL. Compared to skin cancer cells (melanoma—LM-MEL-75 and epidermoid carcinoma—A431), the aqueous extract of *F. betulina* exhibited cytotoxicity at lower concentrations, which were non-toxic to normal human dermal fibroblasts. Notably, the melanoma cell line demonstrated statistically significant cytotoxic potential for the tested extract even at a concentration of 100 µg/mL.

Importantly, the tested extract did not exhibit cytotoxic activity toward normal kidney cells (Vero). This lack of cytotoxicity is particularly significant for drug elimination from the body. In contrast, cisplatin demonstrated cytotoxic potential even at a concentration of 0.1 µg/mL.

Interestingly, up to a concentration of 10,000 µg/mL, we observed increased mitochondrial activity in normal intestinal epithelial cells (CCD 841 CoN). Simultaneously, there was a concentration-dependent inhibition of activity in both HT29 and LoVo colorectal cancer cells. Notably, the LoVo cell line exhibited cytotoxic potential at a concentration of 10 µg/mL.

In the case of lymphoblastic leukemia tumors (CCRF-CEM)—these cells come from lymphoblastic leukemia and express certain receptors such as CXCR4 [18]). The impact of the tested extract was observed only at the highest tested concentration. However, in the case of fibrosarcoma, cytotoxic potential was observed already at a concentration of 100 µg/mL. The tested extract demonstrated a significant impact in the context of lung cancer (A549) at a concentration of 10 µg/mL. No antitumor activity was observed in breast (MCF7) and ovarian (HeLa) cancer cell lines, and there was even an increase in mitochondrial activity.

IC_50_, which refers to the concentration of a substance required to inhibit the growth of 50% of cells, allows for the evaluation of the effectiveness of these substances in inhibiting cell viability. These values were determined for the tested extract and cisplatin in various cell lines, both cancerous and normal (Table 1).

For the *F. betulina* extract, it can be observed that it has relatively low toxicity toward normal dermal fibroblast cells (NHDF) and fibroblasts (L929), with IC_50_ values of 27,409.79 µg/mL and 47,995.60 µg/mL, respectively. Similarly, in the case of the Vero cell line (monkey kidney cells), the IC_50_ is 23,833.79 µg/mL, indicating low toxicity toward normal cells. For the CCD-841 cell line, which represents normal intestinal epithelium, the extract was non-toxic, which is an interesting observation since, for cancerous intestinal lines such as HT-29 and LoVo, the IC_50_ values were 2996.55 µg/mL and 7204.86 µg/mL, respectively. This indicates that *F. betulina* exhibits some selectivity toward cancerous intestinal cells compared to normal intestinal epithelium.

Regarding the Mel75 cell line (melanoma), the *F. betulina* extract showed relatively high cytotoxicity (IC_50_ = 2367.68 µg/mL), which, when compared to the values for normal dermal fibroblast cells (NHDF), indicates significant selectivity toward cancerous cells. This suggests that the extract may be promising in the context of melanoma therapy, as its toxicity to normal dermal fibroblast cells is considerably lower.

On the other hand, cisplatin, a widely used anticancer drug, demonstrated significantly higher cytotoxicity in cancer cell lines. For A431 cells (squamous cell carcinoma of the skin), the IC_50_ was 6.04 µg/mL, and for the A549 cell line (lung cancer), it was 5.02 µg/mL, which reflects its effectiveness in targeting cancer cells. In the case of the colorectal cancer cell line (HT-29), cisplatin showed moderate toxicity (IC_50_ = 13.58 µg/mL), while it was even more toxic to normal intestinal epithelial cells (CCD-841), with an IC_50_ of 3.54 µg/mL.

On the other hand, in cisplatin’s action on melanoma cells (Mel75), the substance was found to be non-toxic, suggesting that cisplatin may not be an effective drug in treating this type of cancer, in contrast to the *F. betulina* extract. Cisplatin also showed no toxicity toward several other cell lines, such as fibroblasts (L929) and osteosarcoma cells (WEHI-164).

In conclusion, the *F. betulina* extract exhibits interesting selectivity toward cancerous cells, particularly in the case of skin and intestinal cancers, while being less toxic to normal dermal fibroblast cells, fibroblasts, and intestinal epithelium. Cisplatin, on the other hand, shows high efficacy against most tested cancer cell lines, especially skin and lung cancers, but lacks activity against melanoma.

#### 2.1.2. SRB Assay

**Figure 2:**
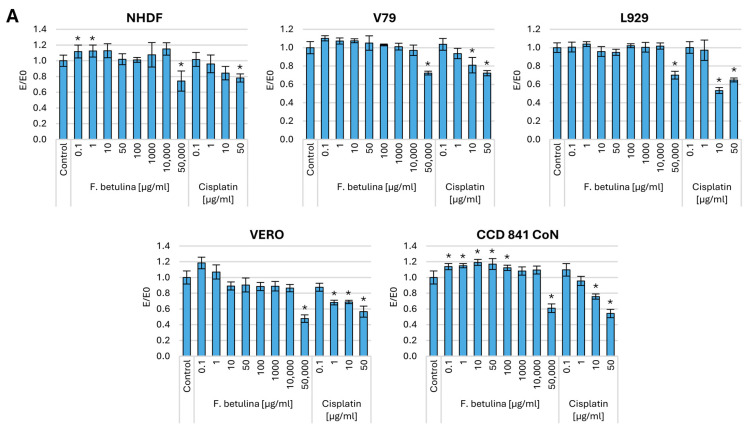

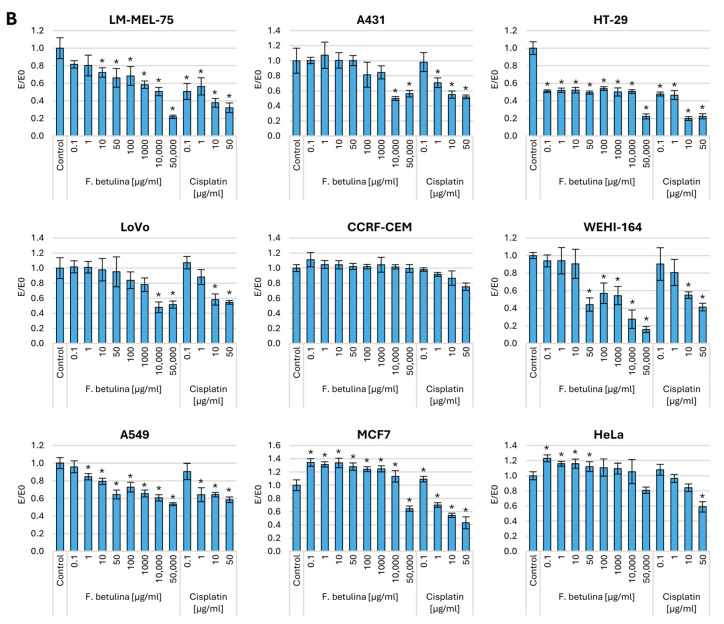
Cell viability after incubation with an aqueous extract of *F. betulina*: (**A**) Normal cell lines; (**B**) Cancer cell lines. Data are presented as E/E_0_ (where E—the average number of colonies in the tested extract concentration and E_0_—the average number in the control culture and standard deviation (SD); * statistically significant differences in cell viability (*p* < 0.05) when comparing treated versus control groups, with error bars representing standard deviation.

To assess the total protein content in cells treated with birch stump aqueous extract, we conducted a sulforhodamine B (SRB) assay. SRB binds stoichiometrically to proteins under slightly acidic conditions, allowing us to measure the overall protein content within the cell. This measurement reflects both the structural and functional activity of the cell. To evaluate the impact of administering the *Fomitopsis betulina* extract, we analyzed cells that had been preincubated with the aqueous extract for 48 h. Additionally, we examined control cells treated solely with medium and a cytostatic agent commonly used in cancer therapy—cisplatin. Figure 2 presents the results of the SRB assay.

Regarding a 48 h incubation of normal cells, both fibroblasts (NHDF, V79, L929) and colon epithelium (CCD 841 CoN) resulted in an increase in the amount of total protein in the culture, which may indicate increased proliferation of normal cells. At the same time, no statistically significant cytotoxic effects were observed in normal kidney cells.

Significantly strong cytotoxicity was observed toward melanoma cells, with statistical significance evident from a concentration of 10 µg/mL. Similarly, in the case of the colorectal cancer cell line HT-29, a potent cytotoxic effect was observed across the entire concentration range, comparable to concentrations of 0.1–1 µg/mL cisplatin. However, cytotoxicity toward A431 and LoVo cells was only evident at the highest tested concentrations. It is worth noting that in the case of hormone-dependent cancer—MCF7 and cervical cancer (HeLa), an increase in the amount of cellular protein (increase in proliferation) was observed. There was no significant effect of the tested extract on lymphoblastic leukemia cells (CCRF-CEM), and there was a strong cytotoxic effect on fibrosarcoma (WEHI-164). In the case of lung cancer (A549), statistically significant cytotoxic effects are observed at levels as low as 1 µg/mL.

#### 2.1.3. Clonogenic Assay

**Figure 3:**
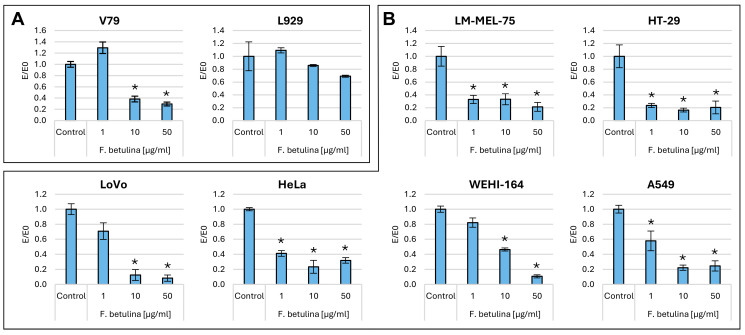
Cell colony formation properties after incubation with an aqueous extract of *F. betulina*: (**A**) Normal cell lines; (**B**) Cancer cell lines. Data are presented as E/E_0_ (where E—the average number of colonies in the tested extract concentration and E_0_—the average number in the control culture and standard deviation (SD); * statistically significant differences in cell colony formation (*p* < 0.05) when comparing treated versus control groups, with error bars representing standard deviation.

The clonogenic test allows for assessing differences in cell ability to produce offspring after treatment with various bioactive compounds [22]. Figure 3 illustrates the colony formation properties of cells following incubation with an aqueous extract of *F. betulina* and cisplatin as a control.

### 2.2. Plant Model Organisms

Cisplatin, within the tested concentration range of 0.1–10 µg/mL, completely inhibited the formation of colonies in all tested cell lines—both normal and cancers. However, the aqueous extract from *Fomitopsis betulina*, tested at concentrations ranging from 1 to 50 µg/mL, did not exhibit any cytotoxic effects on colony formation. Specifically, in the recommended cell line (V79), according to ISO 10993-5 standard [23], there was a statistically significant reduction in colonies at concentrations of 10 and 50 µg/mL compared to the control. Similarly, colorectal cancer cells (LoVo) and fibrosarcoma (WEHI-164) also showed statistically significant reductions in colonies at the same concentrations. Notably, across the entire tested concentration range, melanoma (LM-MEL-75), colorectal cancer (HT29), ovarian cancer (HeLa), and lung cancer (A549) all exhibited statistically significant reductions in colony formation (Figure 3). In the case of breast cancer (MCF7) and epidermoid carcinoma (A431), an increase in the number of daughter colonies was observed after 7 days of incubation, even at the highest concentration tested compared to the control.

#### 2.2.1. Garden Cress (*Lepidium sativum*)

While cress is not directly employed in human cancer research, it serves as a valuable model for investigating biological processes that could have implications for anticancer therapies. For instance, studies on cell cycle regulation, apoptosis, and stress responses may yield insights into potential anticancer compounds [24,25].

**Figure 4:**
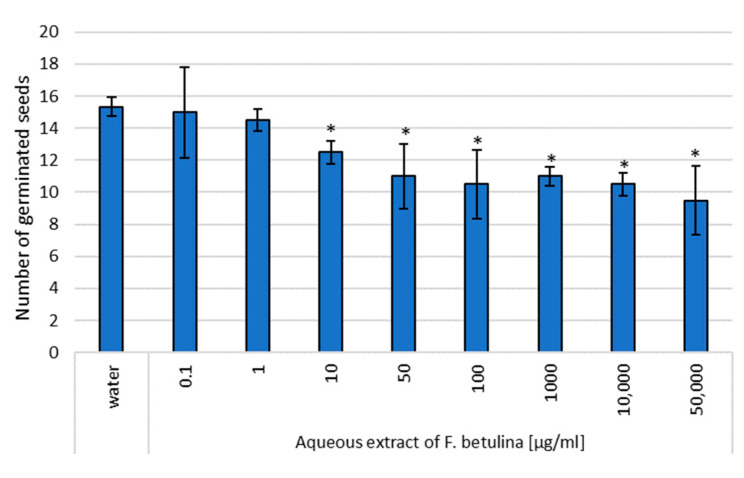
Assessment of the genotoxic effect of an aqueous extract of *F. betulina* on cress seeds; * statistically significant differences in number of germinated seeds (*p* < 0.05) when comparing treated versus control groups, with error bars representing standard deviation.

**Figure 5:**
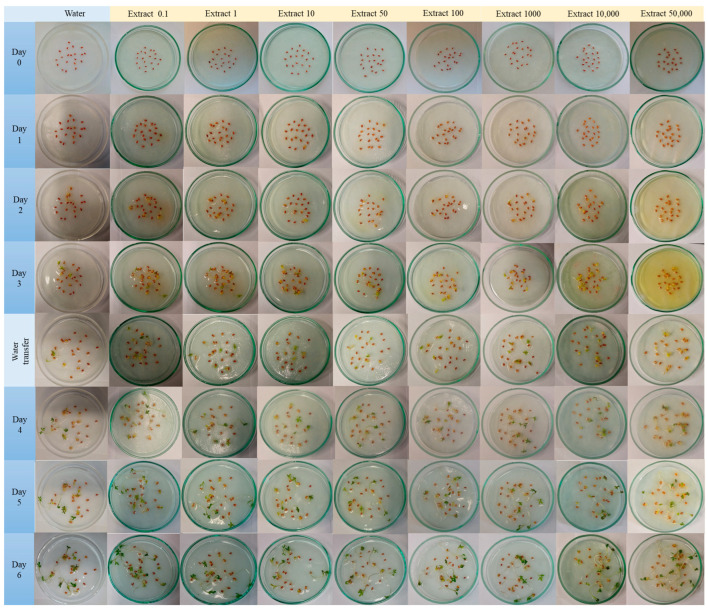
Representative photos from a test of cress during growth and exposure to an aqueous extract of *F. betulina* on cress seeds.

In this study, the aqueous extract of *Fomitopsis betulina* demonstrated statistically significant inhibition of cress germination within the concentration range of 10–50,000 µg/mL. Notably, transferring the seeds to fresh paper soaked only in water did not lead to an increase in the number of germinating seeds (Figure 4 and Figure 5). These findings suggest that the tested aqueous extract containing *F. betulina* may potentially impact apoptosis and cell cycle inhibition.

#### 2.2.2. Modified *Allium cepa* L. Test

Due to the potential impact of the extract on the cell cycle, a commonly used test was performed to assess the disruption of cell division in a plant model organism. In studies on compounds with anticancer activity, assessing the inhibition of the mitotic index in common onion (*Allium cepa* L.) is crucial. The mitotic index serves as an indicator of mitotic activity, representing cell division. In the case of onions, inhibiting this index may suggest potential anticancer effects [26].

**Figure 6:**
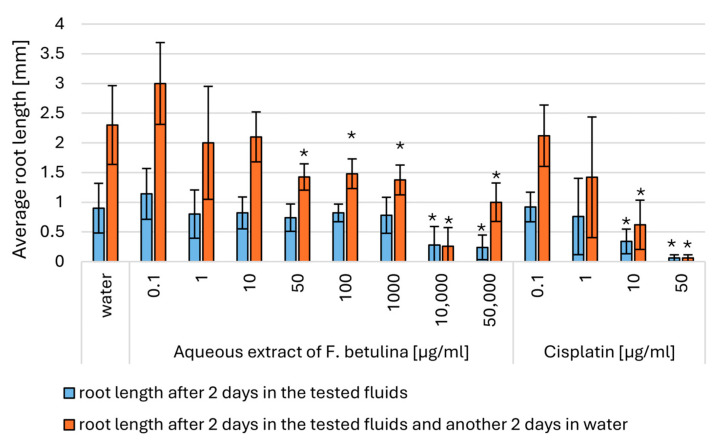
Average root length in Modified *Allium cepa* L. test; * statistically significant differences in average root length (*p* < 0.05) when comparing treated versus control groups, with error bars representing standard deviation.

**Figure 7:**
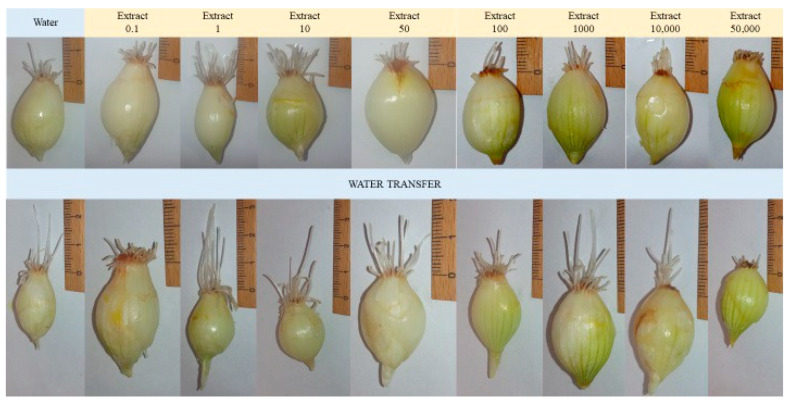
Representative photos from a Modified *Allium cepa* L. test during growth and exposure to an aqueous extract of *F. betulina*.

Direct exposure of onion roots to aqueous extracts of *F. betulina* resulted in a statistically significant reduction in root growth at concentrations of 10,000–50,000 µg/mL. Furthermore, statistically significant prolonged cell cycle arrest was observed at concentrations as low as 50 µg/mL of the aqueous extract (Figure 6 and Figure 7). This indicates that the aqueous extract of *F. betulina* affects the cell cycle, which is relevant both in drug development for cancer therapy and chemoprevention.

## 3. Discussion

Numerous studies have explored the potential anticancer activity of birch tree stump extracts. These investigations primarily focus on analyzing the composition of substances found in water and alcohol extracts. However, their assessment is often limited to specific cancer cell lines, evaluating the cytotoxic effects on individual cell types. Previous research on fungi like *Ganoderma lucidum* and *Trametes versicolor* has highlighted the importance of understanding the broader range of effects that fungal extracts can have on various cancer types, including the selective cytotoxicity toward tumor cells [8,9]. Given the growing interest in understanding the biological activities of natural compounds, especially in the context of anticancer properties, we deemed it crucial to characterize the impact of *Fomitopsis betulina* extract across a broad spectrum of cancer and normal cell lines. Additionally, an essential aspect of our research involves employing model organisms. While cell cultures serve as an initial screening platform for assessing potential bioactive compounds, they do not fully represent complex tissues and organs. Consequently, false positive results may arise. Ethically exploring research with plant model organisms, before resorting to in vivo studies using laboratory animals, holds promise. The study investigated the anticancer activity of an aqueous extract from *Fomitopsis betulina* and explored its potential for further research. Specifically, we examined how this extract affected nine different cancer cell lines. Additionally, we assessed its impact on four normal cell lines to determine the selectivity index. Viability experiments were conducted to analyze the extract’s effects on total protein content (SRB) and mitochondrial activity (MTT). Furthermore, we investigated its influence on cell progeny formation using a clonogenic assay. Finally, we compared the potential mutagenic effects of the extract on cell cycle disruption in a model of common onion roots.

The study of the effect on cells and the anticancer activity of the tested aqueous extract from *F. betulina* was conducted on continuous cell lines. An initial assessment of the toxicity of the tested compounds for non-cancer (normal) cells was performed on L929 and V79 mouse fibroblast cultures, following the recommendations of ISO 10993-5 [23], as well as on Vero kidney cells (normal kidney cells). It is widely known that cytostatics affect the proper functioning of the kidneys. Additionally, studies were carried out on human skin fibroblasts (NHDF) to determine the selectivity index compared to melanoma and epidermoid carcinoma lines. Furthermore, to analyze the selectivity index for colorectal cancer, investigations were conducted on normal intestinal epithelial cells (CDD841). The selection of cancer cell lines was guided by the National Cancer Institute’s guidelines to assess the impact of the tested compounds on various types of cancer. According to the latest World Health Organization (WHO) cancer report, updated in 2020 by the International Agency for Research on Cancer (IARC), the most commonly diagnosed cancers that continue to cause high mortality include lung cancer (A459 lines selected here), colorectal cancer (HT29 and LoVo lines selected here) in women breast cancer (MCF7 cell line) and ovarian cancer (HeLa). Skin cancer (melanoma—LM-MEL-75 and epidermoid cancer—A431) is an equally serious problem. Lymphoblastic leukemia (CCRF-CEM) and fibrosarcoma (WEHI 164) are also a big problem. The incidence of each type of cancer varies according to the region of the world, risk factors, and accessibility to healthcare and screening [27].

The choice of water extract for this study is crucial, as it reflects a safer alternative to alcohol-based solvents, which have been shown to pose carcinogenic risks, according to the World Health Organization (WHO) [28]. Water is a natural biological solvent, making it ideal for in vitro and in vivo testing. It is also safe and non-toxic compared to alcohol. Ethanol, on the other hand, is more effective than water in dissolving many organic compounds and can, therefore, be used to extract specific components [29]. However, the WHO underlines that alcohol (ethanol) is a carcinogen, and there is no safe amount to consume in terms of cancer risk [28]. Therefore, if one wishes to study extracts from natural products in the context of anticancer effects, one should avoid those that may be damaging to the body.

Previous studies on the anticancer activity of *F. betulina* extract and isolated compounds (betulin, betulinic acid, and triterpenes) were carried out using ethanol extract, which resulted in increased cytotoxicity toward melanoma lines (A375, Hs895) and glioma lines (U251MG, U343MG) [8,9]. In comparison to previous studies using ethanol extracts of *Fomitopsis betulina*, which showed strong cytotoxicity toward glioma cells [9], our use of aqueous extracts presents a safer alternative with selective toxicity toward lung and colon cancer cells. This highlights the potential application of aqueous extracts for cancer treatment with lower side effects. Our research shows the effect of *F. betulina* extract on cancer and normal cells. We demonstrated that the aqueous extract had a weaker effect than cisplatin on the tested cancer cells, but there was, statistically, significantly better selectivity toward normal cells compared to the cisplatin effect. At the same time, in the case of the lung cancer cell line (A549) and colorectal cancer cell line (LoVo and HT29), the cytotoxic potential was observed for 24 h incubation.

The chemical composition of *F. betulina* is complex, comprising various bioactive compounds. The main constituents identified in the mushroom are polysaccharides, terpene compounds, fatty acids, and sterols. These compounds contribute to its numerous therapeutic properties such as antimicrobial, anti-inflammatory, antioxidant, immunomodulatory, and anticancer effects [15]. The presence of triterpenes in *F. betulina*, such as betulin and betulinic acid, is consistent with findings in other medicinal mushrooms like Ganoderma lucidum, where similar compounds have demonstrated anticancer properties through apoptosis induction and cell cycle arrest [10,30]. Our own research demonstrated that the tested extract exhibited its most potent effect on the A549 cell line at a concentration of 50 µg/mL for 24h incubation and 1 µg/mL for 48h incubation. However, other researchers examining isolated compounds, including terpene, did not observe any substantial cytotoxic effects [9]. The cytotoxic effects observed in melanoma and lung cancer cells may be attributed to the presence of triterpenes, which have been documented to induce apoptosis through mitochondrial pathways. The lack of activity in breast and ovarian cancer cells suggests hormone dependency, which could limit the extract’s efficacy. Further mechanistic studies could explore the role of specific compounds in modulating the PI3K/AKT and MAPK pathways. These results underline the importance of investigating the synergistic effects of multiple bioactive compounds within the extract rather than focusing solely on individual components.

What is also crucial is the source of our extract: mycelium or fruiting bodies. In the context of A375 and WM795 cells (melanoma), which exhibit varying metastatic potential, we observed a more favorable effect with the mycelium extract. However, the fruiting body extract showed no impact on the WM795 cell line [21]. Our study also revealed the inhibitory effect of the tested mix mycelial and fruiting body aqueous extract on cell growth, irrespective of the incubation time with the extract.

Previous literature reports suggest that the tested compounds from the extract, as well as the fruiting and/or mycelium extract itself, may impact the cell cycle and influence apoptosis in the context of anticancer activity. Consequently, we decided to investigate this potential mechanism using plant model organisms commonly employed for such studies: cress and common onion [26,30]. The use of model organisms like *Lepidium sativum* (cress) and *Allium cepa* (onion) is supported by previous studies on their role in cytotoxicity and genotoxicity assessments, particularly in evaluating natural extracts [30,31]. Garden cress (*Lepidium sativum* L.) serves as a model plant for ecotoxicity assessments in terrestrial ecosystems. This species is particularly suitable for evaluating the cytotoxic effects of tested extracts due to its consistent seed germination and root elongation. These characteristics contribute to reliable and repeatable results, allowing for experimental designs with a limited number of repetitions [24,25]. *Allium cepa*, or common onion, is tested in genotoxicity tests, assessing the inhibition of mitotic division due to several basic features. Onion is characterized by rapid root growth, which allows the genotoxic effect to be observed in a relatively short time. Chromosomal abnormalities can be easily observed under a microscope, making the results easier to view. Moreover, the modified root length growth observation test significantly speeds up the procedure and is also very effective. *Allium cepa* is a popular food plant, which makes it accessible and relatively cheap to study. In short, *Allium cepa* is a universal tool for defining genetic applications [26,31]. After 72 h of incubation, the presence of aqueous extracts had varying effects on the germination of *L. sativum* seeds compared to those germinating in distilled water. No statistically significant germination toxicity was observed in seeds exposed to the lowest two concentrations of the tested extract. However, higher extract concentrations in the medium led to a statistically significant reduction in germination. This observation suggests a potential genotoxic or pro-apoptotic effect of the tested extract. Additionally, inhibition of *Allium cepa* L. root growth occurred upon direct administration of the extract. Interestingly, this effect persisted even after discontinuation of direct administration and subsequent incubation of *Allium cepa* L. in water, indicating an impact on mitotic divisions—the cell cycle.

Limitations: The composition of compounds present in *Fomitopsis betulina* can vary depending on the location and time of collection of this fungus. There are differences in the chemical composition between extracts obtained from fruiting bodies collected from different trees as well as between extracts from fruiting bodies and fungal mycelia.

The location of the mushroom collection can affect the chemical composition due to differences in environmental conditions such as soil type, humidity, sunlight, and the type of host tree [32]. For example, extracts from *Fomitopsis betulina* collected from birch trees growing in different regions may contain different amounts and types of biologically active compounds.

The time of collection also affects the chemical composition of *Fomitopsis betulina*. Differences in weather conditions and developmental stages of the fungus can affect the production and accumulation of different chemical compounds [32]. Although we did not present the chemical composition of the *F. betulina* mushroom in our work, it is worth emphasizing that its composition has been well studied in numerous scientific publications and is relatively constant.

The use of plant model organisms also presents limitations. While it is acknowledged that results from plant model organisms cannot be directly extrapolated to humans, it is important to recognize that these organisms are more complex than single monolayers of cells lacking tissue organization. Furthermore, preliminary research involving plant organisms enables us to investigate specific mechanisms of action without resorting to laboratory animals, which is ethically significant.

The *Fomitopsis betulina* extract has shown promising anticancer properties in in vitro studies, particularly in relation to selected cell lines. However, to assess its potential as an anticancer therapeutic in clinical settings, further in vivo studies and clinical trials are necessary. Such studies would allow for a better understanding of the extract’s bioavailability, metabolism, and potential interactions with other drugs used in cancer therapy. Additionally, it will be crucial to determine the molecular mechanisms of the extract’s action, including its influence on signaling pathways responsible for apoptosis and cancer cell proliferation. Conducting preclinical studies, including animal models, and then clinical trials will enable the evaluation of the extract’s efficacy, safety, and any possible side effects associated with its long-term use.

## 4. Materials and Methods

### 4.1. Raw Material

*Fomitopsis betulina* specimens were collected in October 2021 from the Lower Silesia forests voivodeship near the village of Ławszowa. The mushrooms were carefully detached from the tree trunk to avoid causing damage. Subsequently, the collected mushrooms were cleaned, sliced into 1 cm sections, and weighed. These *F. betulina* slices were then subjected to 72 h of drying in a specialized mushroom dryer. After this drying period, the mushrooms were re-weighed, revealing a weight reduction of approximately 24%. Finally, the dried *Fomitopsis betulina* was ground using a 160 W electric mill.

### 4.2. Extraction Methods

**Figure 8:**
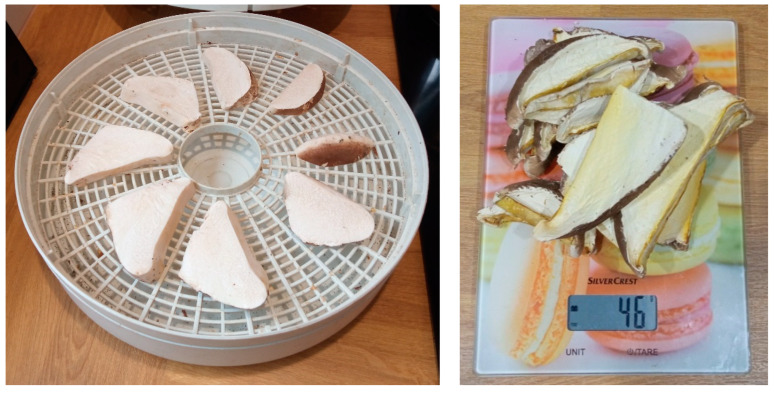
*F. betulina* collected in the Lower Silesian forests and used to prepare the extract came from the same collection.

An amount of 5 g of milled *F. betulina* was weighed and poured over distilled water to reach a final weight of 50g (Figure 8). The mixture was placed on a plate and brought to a boil (95 °C), then it was boiled for 10 min. After this time, the cooled extract was centrifuged at 4500 RPM for 10 min. The supernatant was passed through filters (TPP, Trasadingen, Switzerland; Syringe—filter 0.22 µm, cat. no. 99722).

### 4.3. Tested Compounds

The extract was prepared immediately before the tests. After appropriate dilution of the stock extract, the following concentrations were tested: 0.1, 1, 10, 50, 100, 1000, 10,000, and 50,000 [µg/mL]. The concentration range (from 0.1 µg/mL to 50,000 µg/mL) was selected based on preliminary cytotoxicity studies and literature data, indicating that concentrations within this range can induce effects in various cancer cell lines while maintaining low toxicity toward normal cells. This allows for the assessment of the dose-response relationship and the determination of the therapeutic window. Cisplatin (Merck Sigma, St. Louis, MO, USA, CAS 15663-27-1) was used as a control in relation to the test substance at concentrations of 0.1, 1, 10, and 50 [µg/mL].

### 4.4. Cell Culture and Conditions

The study utilized normal cell lines NHDF, L929, CCD841, V79, and Vero. Also, the compounds were tested on cancer cell lines, including HeLa, WEHI 164, A549, CCRF, MCF7, LoVo, HT29, A375, and A431. All cell lines were maintained in an incubator using special culture bottles containing cell media. For the NHDF line, we used DMEM (cat. no. BE12-917F; Lonza, Basel, Switzerland) without the addition of phenol red. The culture medium for L929, CCD841, MCF7, Vero, HeLa, and A549 lines was EMEM (Eagle’s minimum essential medium) (cat. no. 01-025-1A; Biological Industries, Beit-Haemek, Israel). Lovo cells were maintained on DMEM/F12 (HAM) 1:1 medium (cat. no. 01-170-1A; Biological Industries), while HT-29 cells were cultured in Mc Coy’s 5A Medium (modified) (cat. no. 01-075-1A; Biological Industries). RPMI 1640 medium (cat. no. 01-100-1A; Biological Industries) was used for WEHI 164 and CCRF lines. Lines A375, A431, and V79 were cultured in DMEM high Glucose medium (cat. no. 01-056-1A; Biological Industries). All culture media were supplemented with 10% FBS (fetal bovine serum) (cat. no. P30-8500; PAN Biotech, Aidenbach, Germany), 2 mM L-glutamine (cat. no. BE17-605E/U1, Lonza), 50 mg/mL Gentamycin Sulfate (cat. no. 03-035-1B; Biological Industries), and amphotericin B (cat. no. 15290-026; Gibco, Thermo Fisher Scientific, Waltham, MA, USA). The incubator maintained 95% humidity, a temperature of 37 °C, and a 5% CO_2_, 21% O_2_ concentrations. Tank water in the incubator was enriched with Aquaguard-1 solution (cat. no. 01-867-1B; Biological Industries). Cultures were evaluated at least twice a week using an inverted microscope, and cells were used for testing when they were in the logarithmic growth phase with confluence above 70%. Adherent cell lines were trypsinized using TrypLE solution. The doubling times for the cancer and normal cell lines used in this study were 24–72 h, which were taken into consideration for the timing of the assays.

### 4.5. Viability Assay

The viability assay was conducted following ISO 10993-5:2009 [23], which outlines test methods for assessing the in vitro cytotoxicity. In this case, cultured cells were incubated with the test substance. The following cell lines were used for the test: L929, LM-MEL-75, LoVo, WEHI 164, A549, V79, CCD841, HT29, NHDF, A431, Vero, HeLa, MCF7, and CCRF-CEM. Cells were Seeded in 96-well plates with 10,000 cells per well. The next day, the test substance was applied to the cells after removing the culture medium. Cells were incubated with the compounds for 24 h. The next day, yellow tetrazolium salt 1 mg/mL (Sigma Aldrich, St. Louis, MO, USA, Cas-No: 298-93-1) dissolved in PBS was added to the cells. Insoluble formazan crystals formed as a result of cellular reactions. Formazan crystals are indicative of viable cells. Dead cells or those with impaired metabolism would not produce significant formazan. The plates were placed in a 37 °C, 5% CO_2_, 95% humidity incubator for 2h After incubation, the supernatant was removed, and alcohol—isopropanol (cat. no. 603-117-00-0; STANLAB Sp. z o.o., Lublin, Poland) was added to allow crystals dissolving. Plates were placed on a shaker for 30 min, and absorbance was measured at 570 nm using a microplate reader (Thermo Scientific Multiskan GO, Waltham, MA, USA, type 1510; REF: 51119300).

### 4.6. The Sulforhodamine-B (SRB) Assay

Assessment of total protein using the SRB test is recommended by the NCI (National Cancer Institute). The following cell lines were used for the test: MCF7, CCD841, HeLa, Vero, A431, HT29, NHDF, V79, A549, and CCRF-CEM. Cells were seeded into 96-well plates at 20,000 cells per well. Incubation with the compounds lasted 48 h. The cells were fixed with cold 50% trichloroacetic acid (TCA); for suspension cells, 30% TCA was used in a volume of 20 µL. The plates were incubated at 4–8 °C for 30 h. The plates were rinsed five times under running water to remove any residual acid and allowed to dry at room temperature. An amount of 100 µL of SRB was added for 30 min, and the plates were left at room temperature. Dye residues were removed by rinsing the plates five times with 1% acetic acid using a sprayer and allowed to air dry. After the plates were dry, 100 µL of Tris base solution was added to all wells to dissolve the dye, and the plates were placed in a shaker for 5 min. Absorbance was measured using a microplate reader (Thermo Scientific Multiskan GO, type 1510) at 485, 489 nm, and 565 nm.

### 4.7. Clonogenic Assay

To evaluate colony formation properties following therapy, cells were seeded in dilutions (100 cells) in 6-well plates according to ISO 10993-5 annex B [23]. The following cell lines were used for the test: LM-MEL-75, HT29, HeLa, A549, V79, and L929. The plates were placed in an incubator and left undisturbed for 7 days until colonies were observed in control samples. After incubation, DMEM was removed, and cells were washed with PBS. Cells were fixed with frozen 80% methanol. The wells were then washed with PSB, and the plates were allowed to dry. Staining of the clones was carried out using 0.5% crystal violet for 20 min. Excess stain was removed by washing with water, and the plates were allowed to dry at room temperature. Only colonies visible to the naked eye (>~0.02 cm) were manually counted. The colony counting process was unbiased, as the counter was unaware of the sample identifiers. The resulting data were then plotted.

### 4.8. Garden Cress (Lepidium sativum)

Garden cress is an annual plant belonging to the cabbage family. It serves as a model species due to its rapid growth and high sensitivity to phytophysical extract. A germination test was conducted to evaluate the biological substances. Seeds of garden cress from Toraf company, Barcelona, Sapain (lot number: PL211604336/045TB) were purchased from an online store (www.ogrodniczowiniarski.pl (accessed on 26 June 2023)). These seeds underwent selection to remove any that were damaged, discolored, or differed in size from the others. Each dish was lined with an 80 g/m^2^ filter paper, saturated with the appropriate concentration of test extract and distilled tap water. Twenty garden cress seeds were placed on each dish, which were then covered. The control and test groups were kept in a sunny location without direct light exposure at room temperature. Observations were conducted for 3 days, after which the seeds placed on the test extract were transferred to dishes with blotting papers soaked in tap water. Observations continued for another 3 days. The test extract was prepared on the first day of the experiment and stored in the refrigerator. Throughout the experiment, 3 mL of the appropriate liquid at room temperature was added to each blotting paper daily.

### 4.9. Modified Allium cepa L. Test

**Figure 9:**
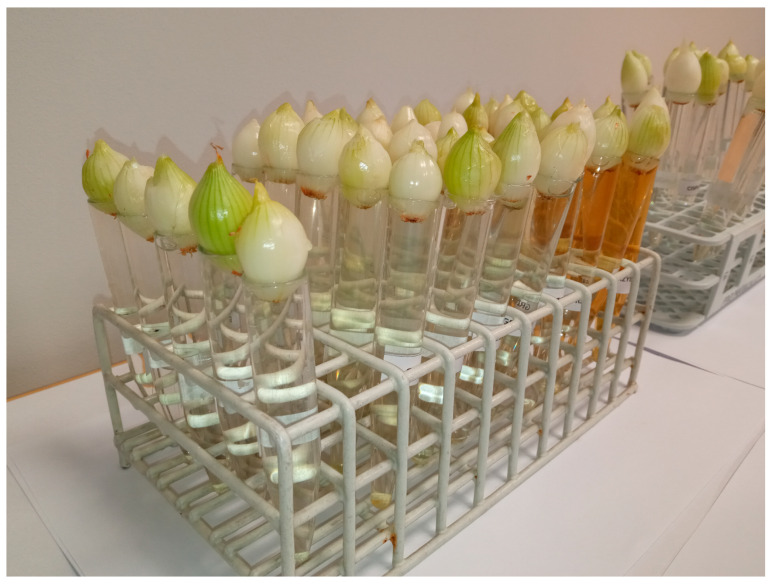
Conducting Modified *Allium cepa* L. test.

The common onion (*Allium cepa*) belongs to the Amaryllidaceae family and is cultivated and consumed worldwide. It is valued for its flavor and health-promoting properties. Depending on the variety, it can be a biennial or annual plant. Onion roots are commonly used in biological tests, such as cytotoxicity and mutagenicity tests. The tests use onion roots, which are often the first to be exposed to chemicals in the environment. The root tip of onions provides results similar to those of animal cytotoxicity tests. The International Program on Plant Bioassays (IPPB) approves onion tests for chromosomal aberrations related to environmental contaminants. Bulbs of spring onions of the Senshyu Yellow winter variety (producer “PIASECKI”, Essington, PA, USA, lot number: PL 14/14/SENS/00006) were purchased. Individuals of similar diameters were selected. Before the test, the scales and the brown pallor were removed from each onion. The root bud (Latin primordium) was left behind. Shriveled, moldy, or spoiled onions, as well as those that had begun to shoot green leaves, were discarded. A modified version of the onion test was used to check the inhibitory effect of the mitotic activity of the tested extracts. The root apices and the base of the onions were placed directly in the test extract at the appropriate concentration and in tap water, bypassing the root germination stage. Five tubers were used for each extract and concentration. Observations lasted 48 h, where two complete mitotic cycles were 30 h (Figure 9). Root evaluation was carried out based on photos taken and length measurements. After this time, the test extract was replaced with tap water for another 48 h. The test extract and tap water were at room temperature. At the end of the experiment, the roots of all bulbs were taken, measured, and evaluated.

### 4.10. Statistical Analysis

Each experiment was performed in triplicate with three biological replicates per assay. The data were reported as the mean ± standard deviation. We assessed the normal distribution and equality of variances using the Shapiro–Wilk and Levene tests, respectively. Additionally, we performed Tukey’s post hoc analysis for ANOVA. The significance level was set at 0.05.

## 5. Conclusions

**Figure 10:**
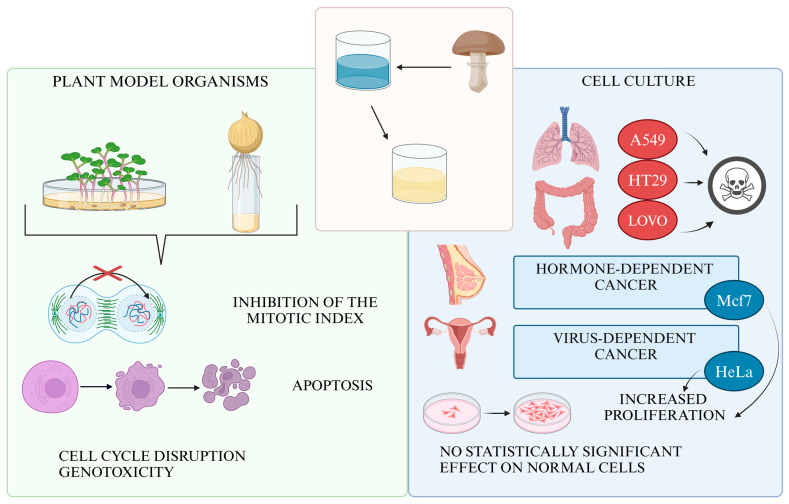
Properties of *F. betulina* aqueous extract in in vitro assays and with model plant organisms.

In summary, our experiments demonstrated that the aqueous extract of *Fomitopsis betulina* exhibits anticancer activity in several cancer cell lines. While its effects were weaker than cisplatin, the extract showed significantly less cytotoxicity toward normal cells. Further research is needed to identify molecular targets and optimize bioactive component extraction. In vivo studies are crucial to assess the extract’s therapeutic potential and safety, especially its effects on metastasis and immune modulation. The extract showed potential activity against lung cancer (A549), colorectal cancer (LoVo, HT29), and melanoma (LM-MEL-75) but had a pro-proliferative effect on hormone- and HPV-dependent cells like breast (MCF7) and ovarian cancer (HeLa). Additionally, it demonstrated selective cytotoxicity, sparing fibroblasts, intestinal epithelium, and kidney cells. Plant model organism studies suggest that the extract may inhibit mitotic divisions, leading to genotoxic and pro-apoptotic effects (Figure 10).

In vitro, the extract acts as a cytostatic rather than a cytotoxic agent on malignant cells. While promising, natural treatments should always be used with caution, complementing rather than replacing conventional therapies.

## Figures and Tables

**Table 1:** The IC_50_ values (µg/mL) for *F. betulina* extract and cisplatin across various cell lines, both cancerous and normal. IC_50_ represents the concentration of a substance required to inhibit the growth of 50% of cells, which allows for the evaluation of cytotoxicity and the potential selectivity of the substance toward cancer cells in comparison to normal cells.

Cell Line	IC_50_ [µg/mL]	
	*F. betulina*	Ciaplatin
NHDF	27,409.79	31.337
V79	22,002.85	>50
L929	47,995.60	20.61
Mel75	2367.68	>50
A431	>50,000	6.04
Vero	23,833.79	>50
CCD-841	>50,000	3.54
HT-29	2996.55	13.58
LoVo	7204.86	>50
CCRF	>50,000	>50
WEHI-164	2175.99	>50
A549	3256.86	5.02
MCF-7	>50,000	20.61
HeLa	16,693.67	8.31

## Data Availability

Raw data are available after contacting the authors by e-mail.
